# Case Report: Micronized purified flavonoid fraction [Daflon]-induced bradycardia

**DOI:** 10.3389/fphar.2025.1612315

**Published:** 2025-08-14

**Authors:** Jhennyfer Aline Lima Rodrigues, Jonathas Pereira das Graças, Gledson de Oliveira Machado

**Affiliations:** ^1^ Universidade Estadual de Goiás, Goiás, Brazil; ^2^ Faculdade de Medicina, Universidade Estadual de Goiás, Goiás, Brazil

**Keywords:** chronic venous disorders, blood pressure, diosmin, flavonoids, hesperidin

## Abstract

**Introduction:**

Flavonoids are oral venoactive drugs frequently prescribed to relieve the symptoms of chronic venous disorders. Micronized purified flavonoid fraction (MPFF, commercial Daflon 1000 mg) is a preparation that contains mainly diosmin and a small fraction of hesperidin. Both active ingredients are flavonoids, known for their anti-inflammatory and antioxidant properties.

**Objective:**

Whether the MPFF can alter hemodynamic behavior was examined.

**Methods:**

Daflon 1,000 mg was administered for 60 days, and the hemodynamic parameters were measured during the circadian cycle.

**Results:**

There were no significant differences for systolic blood pressure (100.1 ± 10.6 mmHg vs. 104.8 ± 7.7 mmHg) or diastolic blood pressure (73.8 ± 3.1 mmHg vs. 76.4 ± 6.6 mmHg) before and after MPFF intake, respectively (p > 0.05). However, in sharp contrast, the heart rate reduced significantly after intake of MPFF (88.8 ± 10.5 bpm vs. 79.3 ± 9.7 bpm; p < 0.05). The mean blood pressure was negatively correlated with the heart rate (HR) in the afternoon (r = −0.450, p = 0.016). During this period, there was a decrease in vein inflammation and varicosity, as shown by magnetic resonance imaging.

**Conclusion:**

We reported a new effect of MPFF as an attenuator of the heart rate. Because venous insufficiency decreased during the observed drug administration period, further studies are needed to examine whether the ability of diosmin and hesperidin to reduce vein disorders is directly linked to their action on heart rate.

## 1 Introduction

The pathogenesis of chronic venous disorders (CVD) is complex and remains incompletely understood. It is already known that the inflammation cycle might reduce the venous tone by modulating changes in cellular components of the venous wall. Deep vein thrombosis (DVT) is a serious medical condition characterized by the formation of a blood clot with a hindering venous reflux mechanism (Huang et al., 2022; Waheed et al., 2025). In addition, varicose veins are tortuous and dilated palpable veins, displaying more than 3 mm in diameter (Segiet et al., 2015). DVT is a common disease in Brazil, and the number of hospitalizations related to venous thromboembolism exceeded 520,000, with more than 67,000 deaths between 2010 and 2019 (Albricker et al., 2022).

Alterations of venous valves occur by a variety of interrelated mechanisms resulting in valve incompetence, structural alterations, and changes in cellular components of the venous wall and can be associated with the effects of contraceptive oral and are accompanied by the risk of thrombosis, mainly considering genetic predisposition (Trenor et al., 2011).

Many types of drugs have various pharmacological actions, notably targeted at the venous wall, and are used to relieve varicose-triggered symptoms, such as swelling, heaviness, and tightness in the legs, or pain in the lower limbs. Micronized purified flavonoid fraction [MPFF - Daflon 1,000 mg] contains diosmin 900 mg plus hesperidin 100 mg and is known for its positive impact on vein inflammation, such as varicose veins and venous insufficiency (Cazaubon et al., 2021).

Both active ingredients of MPFF contain flavonoids, which play anti-inflammatory and antioxidant roles. Diosmin is a synthetic flavonoid derived from hesperidin that acts mainly on the venous system, improving blood flow and circulation, in addition to reducing capillary permeability and preventing the formation of edema. Diosmin also has anti-inflammatory properties, reducing inflammation and pain associated with circulatory problems (Nicolaides, 2003; Bergan, 2005; Huwait and Mobashir, 2022). In addition, diosmin presents benefits by modulating crucial mediators linked to neuroinflammation and neurodegeneration (Singh et al., 2025). In turn, hesperidin is a flavonoid found naturally in citrus fruits, such as oranges and lemons, with antioxidant and anti-inflammatory properties. In the formula, hesperidin acts together with diosmin, with synergic effects, thus helping improve vascular health, preventing and treating circulatory problems such as varicose veins, edema, and venous insufficiency (Huwait and Mobashir, 2022).

Despite the above-mentioned details about the action of MPFF in the venous system, information is very scarce about the impact of MPFF administration on hemodynamic variables, that is, systolic blood pressure (SBP), diastolic blood pressure (DBP), mean blood pressure (MBP), and heart rate (HR). Therefore, the study aimed to verify the influence of micronized purified flavonoid fraction [MPFF - Daflon 1,000 mg] on hemodynamic variables.

## 2 Materials and methods

The data contained in this work were obtained through a review of medical records, an interview with the patient, a photographic record of the diagnostic methods to which the patient was subjected, and a review of the literature. Blood pressure and heart rate measurements were collected daily throughout the treatment, which consisted of 60 days of MPFF - Daflon 1,000 mg intake.

### 2.1 Medical history

R.L.A.J. is a 37-year-old woman, with a BMI of 22.1 kg/m^2^. She had a thrombosis 10 years ago, possibly in association with oral contraceptives and genetic predisposition. After 6 months of treatment with Xarelto, there was a significant improvement in the inflammation. However, varicose veins in the groin area became visible. Recently, persistent dilation of the external jugular vein was described, mainly during strength exercises. Neither the patient nor her relatives present comorbidities, and she describes a sufficiently active lifestyle according to the International Physical Activity Questionnaire (Matsudo et al., 2001). She lives a healthy lifestyle, works, runs twice a week, and does strength exercises three times a week.

### 2.2 Blood pressure and heart rate measurements

Noninvasive systolic (SBP), diastolic blood pressure (DBP), and heart rate were measured in the left arm using a digital manometer (OMRON, HEM-7113, Brazil) after sitting for 5 minutes at rest (Barroso et al., 2020), at least four times a day during the day, including measurements in the morning, afternoon, and evening. The mean blood pressure (MBP) was calculated by the formula: (SBP+2*DBP)/3 (Moran et al., 1995).

### 2.3 Drug administration

The drug intake administration by the patient began immediately after a medical prescription. The recommendation was to take 1,000 mg of MPFF (Diosmin 900 mg plus hesperidin 100 mg) by oral suspension after breakfast for 60 consecutive days.

### 2.4 Medical exams

The patient underwent cardiac ultrasonography (“echo-color-Doppler”) to identify any impairment. A mild tricuspid valve insufficiency was diagnosed. Patients with mild tricuspid regurgitation usually do not require any type of approach directed to the tricuspid valve (Tarasoutchi et al., 2020; Hahn, 2023). The patient also underwent whole-leg and carotid artery ultrasonography (WLUS; also, “echo-color-Doppler”) to seek a resolution for the dilation of the external jugular during strength training or a Valsalva maneuver. The doctor also asked for venous magnetic resonance imaging using MRI technology to image the veins and blood flow of the neck to better confirm the diagnosis.

### 2.5 Statistical analysis

Data normality was tested and confirmed by the Kolmogorov–Smirnov test, allowing the description of values as mean ± standard deviation. Mauchly’s test confirmed the sphericity of the data, enabling the use of parametric statistics for comparisons. Thus, responses during the morning, afternoon, and evening periods were compared using a mixed-model analysis with fixed factors (i.e., “time of response”) and a repeated and random factor (i.e., subject). Student’s t-test was applied to determine the differences between the before and after drug administration time points. Pearson correlation was applied to verify the association between mean blood pressure and heart rate. Statistical Package Sigma Plot software, version 11.0 (Systat Software Inc., 578, Chicago, Illinois) was used, and the level of significance was set at p-value <0.05.

## 3 Results

We first sought to analyze the overall hemodynamic behavior immediately before and at least three times between 60 min and 90 min after [MPFF - Daflon 1,000 mg] administration. These measurements were taken in the morning over 60 days. The SBP, diastolic blood pressure (DBP), and MBP did not change significantly after drug intake over the 60-day analysis (Table 1). However, the HR decreased significantly after intake of MPFF (p < 0.05).

**TABLE 1:** Hemodynamic variables before and after drug administration.

Variables	Before	After	P-value
SBP	100.1 ± 10.6	104.8 ± 7.7	0.112
DBP	73.8 ± 3.1	76.4 ± 6.6	0.321
MBP	82.5 ± 5.0	85.9 ± 6.6	0.130
HR	83.0 ± 10.5	73.3 ± 9.9	0.014*

SBP: systolic blood pressure; DBP: diastolic blood pressure; MBP: mean blood pressure; HR: heart rate. Student’s T. *p < 0.05.

We then examined whether MPFF - Daflon 1,000 mg administration influenced the hemodynamic variables along the circadian cycle. The SBP, DBP, MBP, and HR did not change significantly after drug intake between morning, afternoon, or evening (Table 1, p > 0.05). There was an interval of at least 4 hours between the measurement of variables from the morning to the afternoon and from the afternoon to the evening.

The influence of the day’s circadian cycle on hemodynamic variables, mainly MBP and HR, showed a negative and significant correlation during the afternoon (p < 0.05, Table 3).

**FIGURE 1:**
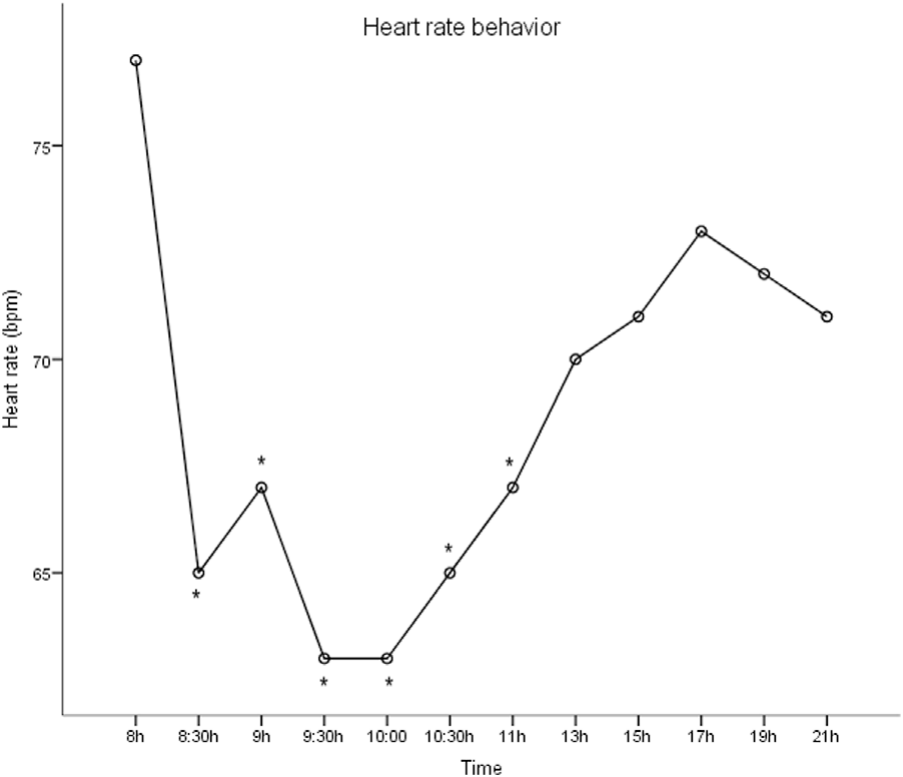
Heart rate behavior during day’s circadian cycle. Mixed-model analysis. *p <0.05.

Figure 1 shows the behavior of the heart rate throughout the day. Before intake of medication, the resting heart rate is higher than it is 30 minutes later (77.7 ± 10.1 bpm vs. 65.7 ± 6.7 bpm, respectively) (p < 0.05). The patient recorded the hemodynamic variables 12 times a day, including measurements in the morning, afternoon, and evening periods. The attenuation of heart rate remains for up to 3 h after intake of the medication (77.7 ± 10.1 bpm vs. 63.7 ± 4.3 bpm, respectively) (p < 0.05).

## 4 Discussion

The aim of the present study was to investigate the behavior of blood pressure and heart rate after 60 days of consecutive use of MPFF. Surprisingly, there is scarcely any information linking hemodynamic behavior with this drug treatment. However, the study is based on a single patient, which limits the generalizability of the findings. For 60 days, we examined whether administration of this drug might influence hemodynamic parameters. The patient described improvement of vein swelling in the neck a few days after drug administration. However, gastrointestinal disorders, such as diarrhea, were reported as an adverse effect that persisted for at least 1 week in the beginning of treatment.

The hemodynamic parameters were monitored over 60 days in a female patient (details subsection Methods/Medical History) and a striking and curious effect of MPFF was found: it triggers a significant decrease in the HR, while it does not alter SBP, DBP, or MBP. The most prominent question regarding the above-mentioned findings is how MPFF can decrease heart rate without changing the SBP, DBP, or MBP.

Note that MPFF is composed of 100 mg hesperidin and 900 mg diosmin. Diosmin contains a naphthalene-derived ring that is very similar to the structure present in the chemical compounds of beta-blocking medications such as propranolol. It is known that α-adrenergic blocking agents have several therapeutic uses, including the relief of several peripheral diseases, such as phlebitis and phlebothrombosis, and that β-blocking agents are mainly used in hypertension and certain arrhythmias (Korolkovas and Burckhalter, 2008).

Alternatively, the chemical structure of diosmin, a flavone glycoside (National Center for Biotechnology Information, 2025), resembles the structure of nebivolol, which increases sympathetic (adrenergic) effects such as increased blood pressure and heart rate. However, the diosmin downstream signaling cascade triggers an inhibitory effect on the sympathetic system, which may explain the bradycardia in the patient reported here in this study. In agreement with this, patients treated orally for 10 days with therapeutic doses of diosmin accumulated and metabolized significantly less noradrenaline than their untreated counterparts. The effects of diosmin consisted of an overall reduction of accumulation of noradrenaline and formation of its metabolites, the latter being more markedly affected than the former (Araujo et al., 1991). Thus, it is plausible to speculate that the diosmin-related decrease in heart rate may be linked to suppression of noradrenaline targets.

In fact, previous studies have reported a clear role for Daflon 500 mg in the treatment of venous disease, either prescribed alone in the early stages of the disease or as a part of the complex management of chronic vein disorders (Nicolaides, 2003; Bergan, 2005; Cazaubon et al., 2021). The protective effects of Daflon address both the macro-circulation and the microcirculation at the same time (Ramelet, 2001). Strictly in terms of blood pressure behavior, there is no significant difference before and after drug intake (Table 1). However, we are aware of this study limitation because all data come from a single patient who had also described a low blood pressure profile before treatment.

Blood pressure (BP) exhibits a circadian rhythm in humans (Douma and Gumz, 2018; Smolensky et al., 2017). As expected, in the present study, there is a normal blood pressure variation during the day without significant differences (Table 2). However, we noted a larger standard deviation in the afternoon period that might indicate greater data variability. In addition, MBP was negatively and significantly correlated with HR in the afternoon period (Table 3). MBP is the pressure that exerts the greatest influence on the self-regulation of blood flow in organs and on the hemodynamic homeostatic mechanisms of the whole body, such as baroreceptors. Because cardiac output is the product of heart rate and stroke volume, changes in either of these parameters also influence MBP. In humans, heart rate at rest is largely under the control of the parasympathetic vagus nerve, while vascular tone is sympathetically mediated (Wehrwein and Joyner, 2013). The greater correlation in the afternoon period could be explained by the circadian influence as well as the peak action of the drug. The systolic and diastolic blood pressure reduced after treatment with diosmin (25 mg/kg, 50 mg/kg, and 100 mg/kg) and an increase in the levels of nitric oxide metabolites (nitrite and nitrate) in male Wistar rats with hypertension induced by deoxycorticosterone acetate salt (Silambarasan and Raja, 2012).

**TABLE 2:** Behavior of hemodynamic variables in different periods of the day after MPFF administration.

Variables	Morning	Afternoon	Evening	P-value
SBP	104.3 ± 7.0	103.2 ± 10.4	104.0 ± 5.1	0.962
DBP	76.6 ± 4.8	75.6 ± 8.0	75.5 ± 3.8	0.468
MBP	85.8 ± 5.1	84.8±	85.0 ± 3.4	0.343
HR	84.0 ± 11.6	77.7 ± 8.7	80.8 ± 7.7	0.075

SBP: systolic blood pressure; DBP: diastolic blood pressure; MBP: median blood pressure; HR: heart rate. ANOVA one-way, p < 0.05.

**TABLE 3:** Correlation between mean blood pressure and heart rate.

Variable	HR					
	Morning		Afternoon		Evening	
MBP	*r*	*p-value*	*r*	*p-value*	*r*	*p-value*
	−0.278	0.160	−0.450	0.016*	−0.162	0.758

MBP: mean blood pressure; HR: heart rate. *Pearson correlation,* *p < 0.05.

## 5 Conclusion

In conclusion, the micronized purified flavonoid fraction - Daflon 1,000 mg induced bradycardia after 60 days of consecutive intake. Apart from that, there is no significant effect on blood pressure. Because venous insufficiency decreased in the observed drug administration period, further studies must examine whether the ability of diosmin and hesperidin to reduce vein disorders is directly linked to their action on heart rate. Confirmation of the findings of this study by further randomized studies might help support this assessment because the study is based on a single patient, which limits the generalizability of the findings.

## Data Availability

The raw data supporting the conclusions of this article will be made available by the authors, without undue reservation.
