# Supplementary material for: Case Report: Micronized purified flavonoid fraction [Daflon]-induced bradycardia
Source: Front Pharmacol. 2025 Aug 14;16:1612315. doi: 10.3389/fphar.2025.1612315 (PMC12390788; doi:10.3389/fphar.2025.1612315)

Supplementary Material

Supplementary Figure 1. External left vein jugular before drug administration.

A)

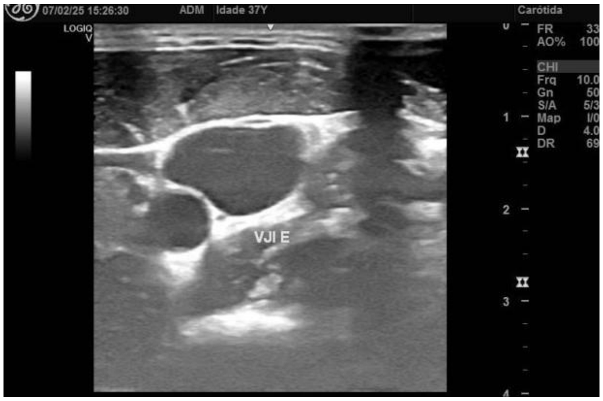

B)

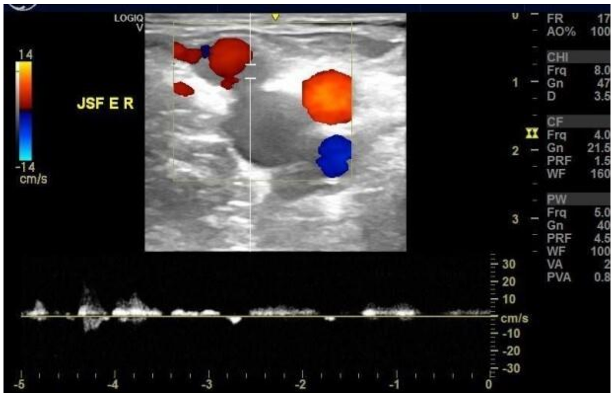

C)

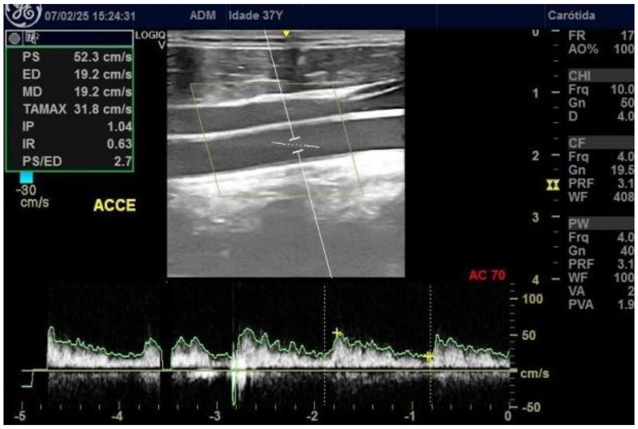

D)

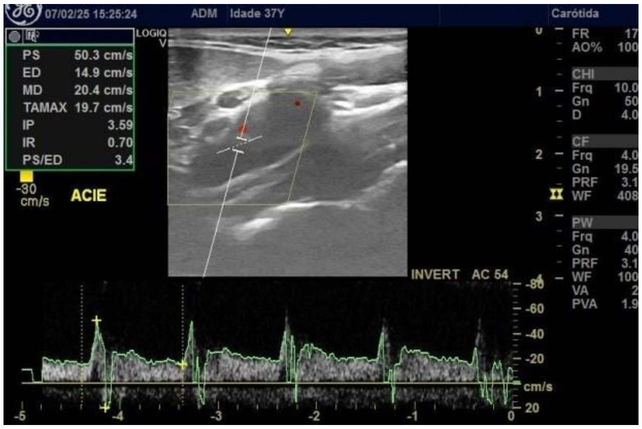

Supplementary Figure 2. External left vein jugular after sixty days of drug administration.

A)

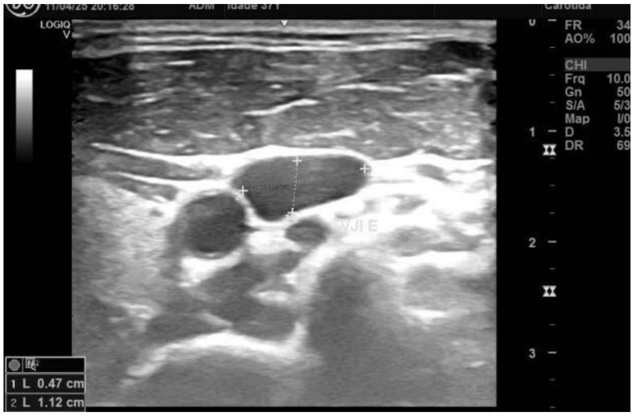

B)

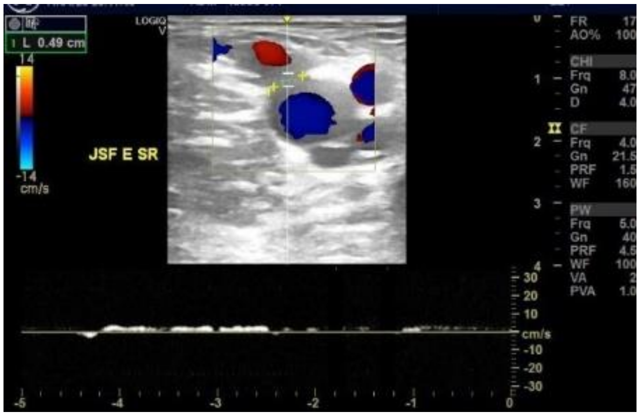

C)

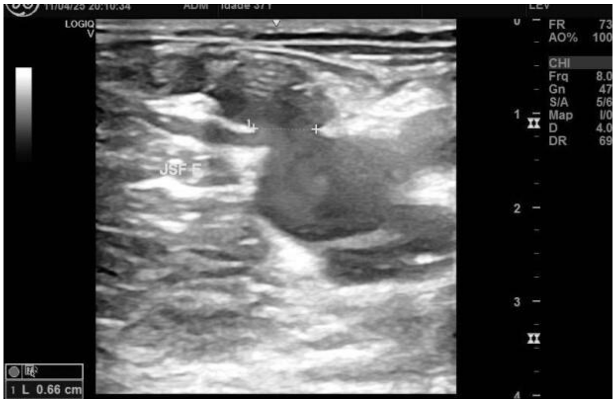

D)

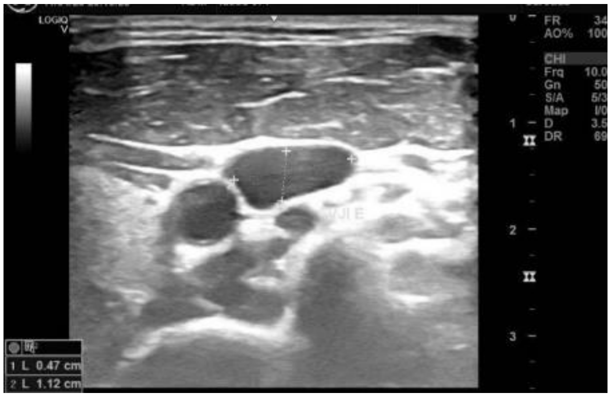

Supplement: Supplementary file 1 [file DataSheet1.pdf]
